# Predicting Blood Lactate Concentration and Oxygen Uptake from sEMG Data during Fatiguing Cycling Exercise

**DOI:** 10.3390/s150820480

**Published:** 2015-08-19

**Authors:** Petras Ražanskas, Antanas Verikas, Charlotte Olsson, Per-Arne Viberg

**Affiliations:** 1 Department of Electric Power Systems, Kaunas University of Technology, Studentų g. 50, Kaunas LT-51368, Lithuania; 2 Intelligent Systems Laboratory, Halmstad University, P.O. Box 823, Halmstad S-30118, Sweden; E-Mail: antanas.verikas@hh.se; 3 Biological and Environmental Systems Laboratory, Halmstad University, P.O. Box 823, Halmstad S-30118, Sweden; E-Mail: charlotte.olsson@hh.se; 4 Swedish Adrenaline, Pilefeltsgatan 73, S-30250 Halmstad, Sweden; E-Mail: pelle.wiberg@hh.se

**Keywords:** blood lactate concentration, cycling, surface electromyography, oxygen uptake, random forest, ridge regression

## Abstract

This article presents a study of the relationship between electromyographic (EMG) signals from vastus lateralis, rectus femoris, biceps femoris and semitendinosus muscles, collected during fatiguing cycling exercises, and other physiological measurements, such as blood lactate concentration and oxygen consumption. In contrast to the usual practice of picking one particular characteristic of the signal, e.g., the median or mean frequency, multiple variables were used to obtain a thorough characterization of EMG signals in the spectral domain. Based on these variables, linear and non-linear (random forest) models were built to predict blood lactate concentration and oxygen consumption. The results showed that mean and median frequencies are sub-optimal choices for predicting these physiological quantities in dynamic exercises, as they did not exhibit significant changes over the course of our protocol and only weakly correlated with blood lactate concentration or oxygen uptake. Instead, the root mean square of the original signal and backward difference, as well as parameters describing the tails of the EMG power distribution were the most important variables for these models. Coefficients of determination ranging from $R^{2}=0.77$ to $R^{2}=0.98$ (for blood lactate) and from $R^{2}=0.81$ to $R^{2}=0.97$ (for oxygen uptake) were obtained when using random forest regressors.

## 1. Introduction

Since the invention of bicycles in the 19th century, cycling has become a significant part of everyday life. It is a popular mode of transportation for short-to-medium distances, as well as a recreational activity. It is promoted by ecology activists as an environmentally-friendly, inexpensive and convenient alternative to fossil fuel-based vehicles. It is also a beneficial exercise, which improves fitness and cardiovascular health, while having a relatively low impact upon leg joints compared to jogging [1], as well as being a popular professional sport.

Fatiguing training sessions are beneficial for building up muscle power, raising lactate threshold and improving maximal oxygen consumption [2]. However, excessive fatigue with limited recovery can lead to over-training and, consequently, increased susceptibility to injuries, which may have a long-term negative impact on an athlete’s career [3,4]. Therefore, establishing methods that evaluate fatigue in a non-invasive manner and that adjust the training regimen accordingly to improve its quality, as well as to avoid the risk of overuse injuries is very important. However, this is a complicated task: fatigue is a phenomenon that has not only physical, but also psychological factors, making subjective assessment of fatigue, such as Borg’s Rating of Perceived Exertion (RPE) scale [5], unreliable; motivated athletes would be willing to continue the task much longer than unmotivated ones.

There are a number of known techniques that can be used to monitor muscle activity non-invasively during an exercise, including, but not limited to, surface electromyography (sEMG), sonomyography (SMG), near-infrared spectroscopy (NIRS) and mechanomyography (MMG) [6]. Of these, sEMG seems to be the most prevalent due to the relative ease of obtaining the signals compared to other modes.

It has been established for over a century [7] that during a sustained static muscle contraction of medium to high force, the spectral components of an sEMG signal are compressed towards the lower frequencies. However, the exact physiological mechanism of muscle fatigue has been explained only relatively recently. As long as the respiratory and cardiovascular systems can deliver oxygen, aerobic respiration is the primary means of converting nutrients into energy. However, once the supply of oxygen is no longer maintained, less effective anaerobic glycolysis dominates as a means of energy production. During this process, lactic acid is accumulated in the working muscle cells. The hydrogen ions contribute to the lower pH of interstitial fluid, which, in turn, reduces the propagation velocity of the action potential along the muscle fibers [8]. Thus, it has become common to monitor levels of blood lactate, as well as oxygen consumption ($VO_{2}$) during exercise as biochemical indicators of anaerobic glycolysis and, indirectly, fatigue.

During a static load exercise, the generated sEMG signal can be assumed to be a result of a wide-sense stationary (WSS) random process, which allows using established techniques, like autoregressive modeling or the short-time Fourier transform (STFT) to observe the compression of the spectral components. A common way to track this effect is obtaining median power frequency (MPF), which decreases as the frequency content shifts to the lower range. This, however, is not feasible for dynamic activities, such as cycling, because the frequency content of the sEMG signal is altered by variations in muscle force and muscle length [9]. Additionally, movement of the muscles under the skin can cause a displacement of the electrodes in relationship to muscle fibers, which may influence the frequency content of the signal unpredictably. This means that techniques designed for the analysis of non-stationary signals should be used instead [10].

Karlsson *et al.* [11] in 2000 compared four time-frequency analysis techniques, the continuous wavelet transform (CWT), the Wigner–Ville distribution, the Choi–Williams distribution and the short-time Fourier transform (STFT), for estimation accuracy and precision of time-dependent spectral moments, including mean, standard deviation, skewness and kurtosis. All four techniques showed very similar results, although CWT was the most, while STFT was the least precise and accurate. However, it must be also noted that STFT is computationally much less complex than CWT.

However, in 2001, Bonato *et al.* showed [9] that for periodic dynamic tasks, it is possible to avoid a lot of complicating factors by assuming the cyclic stationarity of the signal. This assumption is valid, if the following conditions are true:

1. The task is mechanically repeatable in a fairly precise manner, *i.e.*, the trajectory of the task is fixed;
2. The EMG signal is partitioned in such a way as to ensure that only the segments, corresponding to the same phases of a task, are compared;
3. While not strictly necessary, the results can be further improved by averaging over a few such segments to diminish the variance.

In 1998, Jammes *et al.* investigated correlations between the parameters of an EMG of vastus lateralis (VL) muscle, oxygen uptake and blood lactate concentration during a cycling exercise [12]. They showed that the root mean squared (RMS) amplitude of the EMG signal was very strongly correlated to the measured workload of the muscle. Oxygen uptake increased sharply at the beginning, but later on, it would level out; as a result, the $VO_{2}/RMS$ ratio could reach as much as 150% of its initial value by the end of the second minute of the exercise and then drop back to normal or even dip below the initial value during the recovery period after the exercise. The rates of change in this ratio were found to be in correlation with both peak values of $VO_{2}$ and blood lactate concentration.

In 2002, Pringle and Jones [13] hypothesized that maximal lactate steady state, critical power and electromyographic fatigue threshold were all equal. They also aimed to show that continuous work at levels above maximal lactate steady state caused an increase in oxygen uptake and integrated EMG. Results showed that the investigated measures were strongly correlated, but not equivalent; critical power was always significantly higher for all test subjects. When tested at power levels about 20 W above maximal lactate steady state (but still below critical power), all test subjects exhibited an increase in blood lactate concentration and oxygen uptake, suggesting that the maximal lactate steady state is the boundary for the heavy exercise domain. Most significant to our study is the fact that the change in integrated EMG was insignificant in almost all cases.

The conclusions of Pringle and Jones [13] may be further supported by results from Tenan *et al.* in 2011, who investigated a relationship between blood lactate, blood potassium and MPF of the EMG signal of VL during a progressive cycling exercise [14]. Every test subject repeated the protocol twice, once normally and once while in a glycogen-reduced state. In both cases, potassium concentration increased to similar levels. Lactate concentration also increased in both cases, although not as much as in the glycogen-reduced state. However, in either case, they were not correlated strongly with MPF, although for five out of eight subjects, MPF did decrease with time. The authors make the conclusion that local fatigue of VL muscle is not the limiting factor on the duration of the exercise; rather, it is the incapacity of the cardiovascular system to provide muscles with enough oxygen.

Another study of interest is one by Bercier *et al.* done in 2009 [15]. While it was concentrated on all-out sprints and, thus, did not concern fatigue, it found that during the first seven cycles of acceleration from a stationary position, the MPF of EMG recorded from VL increased with cycling velocity. The authors suggest that this is due to differentiated employment of motor units in VL. A similar result was obtained in 2003 by Linnamo *et al.* [16] while investigating an explosive power exercise on leg press equipment. The authors observed that the MPF for VL increased during the exercise and was higher when movements were faster.

While research on fatigue during cycling is mostly concentrated around the VL muscle, some studies also incorporated other muscles. For example, in a study published in 2003, Knaflitz and Molinari investigated changes in EMG of rectus femoris (RF), biceps femoris (BF) and gastrocnemius (GC), as well as coordination of their activation patters, during a 30-min constant-load cycling exercise [17]. Despite test subjects reporting fatigue, the study showed no significant changes to the instantaneous frequency of EMG signals nor muscle activation patterns. It should be noted that the study was conducted at relatively low power levels.

In 1996, Jansen *et al.* measured the systematic effect of blood lactate concentration on the median power frequency of EMG signals taken from both exercising (VL) and non-exercising (flexor digitorum superficialis, FDS) muscles [18]. It was assumed that the MPF would decrease for both muscles as byproducts of anaerobic glycolysis were spread through blood. The results based on 12 subjects were opposite from what was expected, with the MPF in VL increasing for eight subjects out of 12 during the exercise. Only three subjects showed a decrease in median frequency, and one subject had no significant changes at all. Moreover, for all subjects, FDS did not show any changes, suggesting that any chemical substance that would affect the characteristics of EMG signals is not transported to other muscles in substantial amounts.

In contrast, in the year 2000, Gerdle *et al.* used an iso-kinetic knee extension exercise to validate EMG mean frequency and RMS as fatigue indicators for that type of dynamic exercise [19]. Twenty one healthy volunteers performed 100 iso-kinetic knee extensions, and EMG signals were recorded for VL, RF and vastus medialis (VM) of the right thigh. The peak torque was used as an indicator for fatigue. The study found that the mean frequency of EMG was strongly correlated to the peak torque and, thus, provided a good indication of the state of fatigue. No such correlations were found for the RMS of the signal.

In 2008, Dingwell *et al.* performed a series of studies on the changes of muscle activity and the kinematics of highly-trained cyclists during fatigue [20]. Kinematic data and EMG signals from four muscles—VL, tibial anterior (TA), BF and GC—were collected throughout the exercise of cycling at 90 RPM at their ${VO_{2}}_{max}$ power level. The study determined significant correlations between changes in kinematics and EMG, and a reduction in MPF was found in 18 out of 28 muscles tested, most notably in BF and GC.

There is a discrepancy between the findings of various studies whether the spectral characteristics of EMG signals are indicative of the general body fatigue during cycling exercise. Thus, the main aim of our research was to investigate if characteristics of the EMG power spectrum lend themselves to building accurate models from experimental data to predict physiological indicators, namely blood lactate concentration and oxygen uptake, as well as which parameters of the power spectrum are the most informative. For this purpose, multiple statistical characteristics of the power distribution across various frequencies in the spectra of sEMG signals were considered, and both linear and non-linear models were explored. A variety of muscles have been studied by other researchers throughout the years with mixed results; therefore, we included four muscles, considered important in bicycling: vastus lateralis, rectus femoris, biceps femoris and semitendinosus (ST).

## 2. Materials and Methods

### 2.1. Testing Protocol

For each of the test subjects, their ${VO_{2}}_{max}$ and blood lactate threshold were determined several weeks before the main experiments. The subjects were asked to maintain constant cadence at 90–100 rpm throughout the experiment. A blood sample was obtained at the end of each minute. The experiment was divided into three distinct phases:

1. Six minutes of cycling at 60% of ${VO_{2}}_{max}$ power.
2. Cycling at 90%–95% of ${VO_{2}}_{max}$ power until the test subject was unable to maintain the required cadence.
3. Six minutes of cycling at 60% of ${VO_{2}}_{max}$ power.

For test Subjects 7 and 8, the first and third phases were different, both including 5 min of cycling at 50% of ${VO_{2}}_{max}$ and a further 5 min of cycling at 75% of ${VO_{2}}_{max}$. Since in this experiment, only direct moment-by-moment relationships between EMG and physiological indicators are investigated, we do not consider this difference in protocol execution to be significant for our purposes.

### 2.2. Volunteers and Equipment

Nine volunteers (4 women, 5 men, aged 18–56 (average 37), height 158–189 cm (average 174 cm), weight 56–90 kg (average 71 kg)) participated in the experiments with full written informed consent and ethical approval of Regional Ethics Committee in Lund (Regionala Etikprövningsnämnden Lund; Reg.No. 2014/162). The experiments were conducted on a stationary bicycle ergometer (Monark, Vansbro, Sweden). Throughout all of the tests, the following measurements were taken:

- EMG signals were obtained from VL, RF, BF and ST muscles of both left and right legs using bipolar surface electrodes (BlueSensor, AMBU, Copenhagen, Denmark) connected to an eight-channel EMG recorder (Muscle Tester 6000, Megawin, Kuopio, Finland) at a sampling rate of 1000 Hz. The skin was shaved and cleaned with a 0.5-mg/mL solution of chlorhexidine (Fresenius Kabi, Bad Homburg, Germany) and was let to air dry for 1 min before application of electrodes. EMG cross-talk was minimized by placing the electrodes within the border of the specific muscle and with a center-to-center inter-electrode distance of 22 mm [21].
- Oxygen uptake ($VO_{2}$) was measured every 10 s throughout the tests using Jaeger Oxycon Pro (CareFusion, San Diego, CA, USA) and proprietary software supplied with the machine.
- Blood samples were collected every minute using finger prick and analyzed using LactatePro2 (Arkray Europe B.V., Amstelveen, The Netherlands).

Due to technical issues with the hardware, oxygen uptake measurements were unavailable for Subjects 1, 3 and 9.

Volunteers were also asked to rate their perceived exertion on the 6–20-point Borg RPE scale. However, these data were not used, because they appeared to closely follow $VO_{2}$ measurements, which were sampled more frequently, with higher numeric precision, and were not affected by subjective biases.

The collected data were analyzed off-line using custom-written Java 7 (Oracle, Redwood City, CA, USA) applications for data segmentation, combined with MATLAB R2012b (MathWorks, Natick, MA, USA) for statistical analysis.

### 2.3. Signal Preprocessing

The sampled sEMG signals were filtered to remove electronic noise, as well as motion artifacts using Butterworth filters [22]. To remove the electronic noise and other high frequency content from the signal, a 10th order 400-Hz low-pass filter (450-Hz stop band with at least −60-dB attenuation) was used. The signal was then processed further through a 10th order 20-Hz high-pass filter (10-Hz stop band with at least −60-dB attenuation) to remove motion artifacts. The parameters were selected per recommendations set out in other works [23]. The filtered signal is referred to as S(t) in the following text.

The physiological data were interpolated where necessary using Hermite cubic splines with Catmull–Rom tangents [24].

### 2.4. Segmentation of EMG Signals

To ensure that comparisons are made between the same phases of the pedaling task, the filtered sEMG signals were segmented into cycles, each corresponding to a single revolution of bicycle pedals. For that, the following algorithm was applied. First, the backwards difference ΔS(t) of the filtered signal S(t) as described in the previous subsection was obtained:

(1) ΔS(t)=S(t)−S(t−1)

Then, assuming a window length of *N* samples, a function V(t) , characterizing the signal variability in the window, could be defined as follows:

(2) $$V\left(t\right)=\sum_{\tau =t}^{t+N-1}\left|\Delta S(\tau )\right|$$

It must be noted, that the raw signal S(t) could also be used in this definition, basing segmentation on the signal amplitudes instead of variability. However, the variability approach gave more robust and consistent results.

For the purpose of our study, the window length *N* was selected to be 256 samples (256 ms) long, approximately matching a third of a single pedal cycle.

Once the variability function V(t) is established, it can be determined if any particular point in time is the beginning or the end of an activity burst by comparing the variability of windows of equal length before and after the point in question:

(3) $$V_{com}\left(t\right)=\frac{V(t-N)}{V(t)}$$

**Figure 1:**
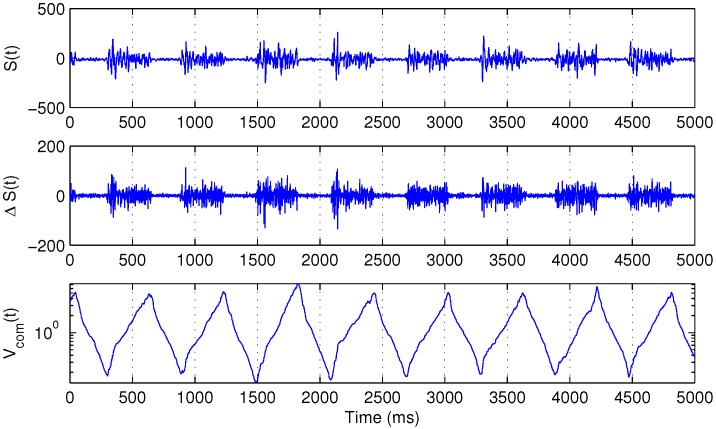
Several bursts of the surface electromyography (sEMG) signal S(t) (**top**), its backward difference ΔS(t) (**middle**) and the corresponding $V_{com}\left(t\right)$ function, defined in Equation (3) (**bottom**).

If *t* is the initial moment of a burst, the function $V_{com}\left(t\right)$ will reach its local minimum. Similarly, if *t* is the end of a burst, the same function will reach its local maximum. Figure 1 illustrates the behavior of $V_{com}\left(t\right)$.

For the purpose of further analysis, first, 256 samples of the raw filtered signal S(t) after each local minimum of $V_{com}\left(t\right)$ were extracted.

### 2.5. Characterizing the Power Distribution

The segmentation performed in the previous subsection allows us to perform calculations that are essentially equivalent to a non-overlapping STFT. Normally, the STFT requires the signal to be WSS in its analysis window. This assumption is probably too strong for windows that are 256 ms long, as cycling involves non-isometric voluntary muscle contractions of changing force. However, based on the fact that cycling is a mechanically-repeatable task, our segmentation algorithm allows assuming cyclic stationarity instead (as discussed in paragraph 7 of Section 1), that is we expect muscle activity to follow the same pattern in each extracted segment. Therefore, while the extracted spectral information from these segments is likely to be affected somewhat by the non-WSS nature of the signal, these effects should be consistent between segments. This, in turn, means that the spectral information is comparable between different segments, and any expected changes, such as energy compression towards lower frequencies, should be observed unhindered.

An alternative way to tackle the stationarity issue could be using any of the time-frequency domain techniques outlined by Karlsson *et al.* [11] or simply shortening the analysis window considerably. However, the latter solution necessarily comes at a cost of lost frequency resolution due to Gabor’s uncertainty principle of signal analysis [25].

To minimize spectral leakage, the segments were multiplied element-wise by the Hamming window function [26] before performing any further operations. Then, for each extracted segment of the signal $S_{i}\left(t\right)$ (where *i* denotes the ordinal number of the segment), a power distribution over different frequencies $D_{i}\left(f\right)$ was calculated by taking the power spectrum $P_{i}\left(f\right)$ (Equation (4)) of the segment and normalizing it to enclose a unitary area (Equation (5)).

(4) $$P_{i}\left(f\right)=\left|DFT(S_{i},f)\right|$$

(5) $$D_{i}\left(f\right)=\frac{P_{i}\left(f\right)}{\int_{0}^{f_{Nyq}}\;P_{i}\left(\overset{˰}{f}\right)\;d\overset{˰}{f}}$$

where $f_{Nyq}$ is the Nyquist frequency (500 Hz for our experiments).

A number of properties defining the segment of the signal were calculated. While usually, this is limited to one or a few properties, such as median power frequency or instantaneous frequency, the set of selected input variables for this research was much larger:

- Time-domain features—root mean square of the signal (RMS, 1) and its backward difference (dRMS, 2), instantaneous frequency (defined as the number of zero-crossings in a given segment of EMG, divided by two) (IF, 3)—were obtained from $S_{i}\left(t\right)$;
- Standard moments of the power distribution across frequencies given by $D_{i}\left(f\right)$—mode (ModF, 4), mean (MnF, 5), standard deviation (StD, 6), skewness (Skew, 7) and excess kurtosis (Kurt, 8);
- Every tenth percentile of the above distribution ($q_{0.1}$–$q_{0.9}$, 9–17), in particular, median power frequency (MPF, 13);
- Relative power contained in 23.44 Hz-wide frequency bands of the power spectrum with 11.72-Hz overlaps, starting with 23.44–46.88 Hz and ending with 234.4–257.8 Hz ($p_{23-47}$–$p_{234-258}$, 18–36), see below for an explanation.

Frequency bands were selected this way, as 23.44 Hz was the width of 6 DFT bins, and the relative power was calculated by summing the contents of these DFT bins with an offset of 3 bins between each measure.

To avoid the inherent variability of EMG signals during the dynamic exercise and to make sure that general trends are not masked by noise, each input variable was passed through an 11 data-point-long sliding window (approximately equivalent to 9–10 s of experiment time) median filter.

The defined input variables were then used to predict blood lactate concentration and oxygen uptake.

### 2.6. Modeling Relations between Input and Output Parameters

The 36 input and 2 output variables, defined in the previous subsection, were used for modeling the relations between them and determining the strongest connections between them using techniques described further.

#### 2.6.1. Spearman’s Rank Correlation

Spearman’s rank correlation coefficient was used to determine whether certain input variables had any significant monotonic trends in relationship with blood lactate concentration and oxygen uptake. Given the ranks of an input variable ${\tilde{x}}_{i}$ and corresponding ranks of an output variable ${\tilde{y}}_{i}$, Spearman’s rank correlation coefficient can then be obtained from this formula:

(6) $$r_{s}=1-\frac{6\sum_{i}{\left({\tilde{x}}_{i}-{\tilde{y}}_{i}\right)}^{2}}{n\left(n^{2}-1\right)}$$

$r_{s}$ is equal to 1 if the relationship between $x_{i}$ and $y_{i}$ is purely monotonically increasing, −1 if the relationship is purely monotonically decreasing and in between otherwise.

#### 2.6.2. Linear Regression Models

Given a set of vectors $x_{i}={\left(x_{i1},x_{i2},...,x_{iN}\right)}^{T}$ of z-scores of input variables and a corresponding set of z-scores of an output $y_{i}$, the linear regression model for predicting the output $y_{i}$ is given by:

(7) $$\overset{˰}{y_{i}}=\sum_{j=1}^{N}x_{ij}{\text{β}}_{j}={x_{i}}^{T}\text{β}$$

The weights ${\text{β}}_{j}$, when using the ordinary least squares method, are estimated in a manner that minimizes the sum of squared error of predictions $\overset{˰}{y}$:

(8) $$\sum_{i=1}^{N}{\left(y_{i}-{x_{i}}^{T}\text{β}\right)}^{2}\to min$$

These estimates of weights are unbiased; however, their variance is high, especially if the input variables are multicollinear, which may cause excessive values of ${\text{β}}_{j}$. Since this was expected to be the case in our study due to the relatively large number of variables in the model, Tikhonov’s (ridge) regularization [27] was employed to counter the multicollinearity effects:

(9) $$\sum_{i=1}^{N}{\left(y_{i}-{x_{i}}^{T}\text{β}\right)}^{2}+\lambda \sum_{j=1}^{P}{\text{β}}_{j}^{2}\to min$$

λ is the shrinkage parameter and is selected in such a way as to minimize the sum of bias and variance, which is estimated using a 10-fold cross-validation technique.

Since all of the variables were used via their z-scores, the impact of a particular input variable on the output can be estimated simply by the value of the corresponding weight ${\text{β}}_{j}$.

#### 2.6.3. Random Forest Nonlinear Models

A random forest (RF) [28] is a general data mining tool, used successfully in different research fields for both classification and regression [29]. Random forests have multiple desirable properties, among them robustness to overfitting, robustness to multicollinearity, robustness to outliers, invariance to monotonic transformations and good handling of mixed-type data. In practice, random forest models show similar performance to support vector machines, which is another widely-used classification and regression technique [29].

**Figure 2:**
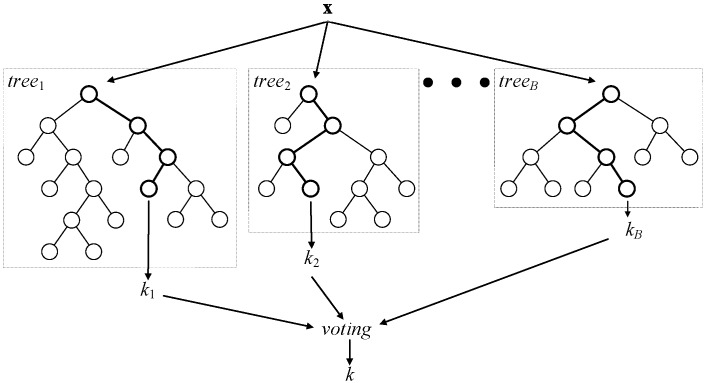
A general architecture of a random forest.

Classification random forest is a committee of decision trees, as shown in Figure 2. In regression random forests, combination by voting is replaced by averaging. To achieve a low correlation of trees, two-step randomization is applied:

1. Each tree of the RF is grown on a bootstrap-aggregated (bagged) sample of the training set. About one third of the samples in the training set do not end up in this sample and, thus, form the out-of-bag dataset for that particular tree. The out-of-bag dataset can then be used for evaluating model performance and variable importance, among other things.
2. When growing a tree, at each node, *n* variables are randomly selected out of the *N* available. At each node, only one variable, providing the best split, is used out of the *n* selected.

As the number of trees in the random forest increases, out-of-bag dataset error decreases and converges to a limit, after which adding further trees no longer improves the performance of the random forest. Moreover, by randomly permuting the values of a certain variable, the out-of-bag dataset can be used to estimate variable importance: variables, for which the deterioration of prediction accuracy due to random permutation is larger, are assumed to be more important for the built random forest model.

In this study, we use an RF of 100 trees, and the number of variables considered at each node split was one third of the total number (as recommended by Breiman in his guidelines to using random forests [30]), *i.e.*, 12.

#### 2.6.4. Assessing Model Performance

The coefficient of determination ($R^{2}$) was used to assess the performance of the regression models. The coefficient is a normalized measure of regression quality comparing the regressor with a perfect regressor ($R^{2}=1$) and a naive regressor (the mean value of teaching data, $R^{2}=0$):

(10) $$R^{2}=1-\frac{\sum_{i}{\left(y_{i}-{\overset{˰}{y}}_{i}\right)}^{2}}{\sum_{i}{\left(y_{i}-E\left[y\right]\right)}^{2}}$$

where *y* and $\overset{˰}{y}$ stand for the actual values and the model prediction, respectively. The $R^{2}$ values presented in this paper represent the average of values obtained through 10-fold cross-validation (ridge regression, independent of 10-fold cross-validation used to obtain an optimal value for shrinkage parameter λ) or 10 different random seeds (random forests).

## 3. Results

### 3.1. Time Course of the Physiological Parameters

**Figure 3:**
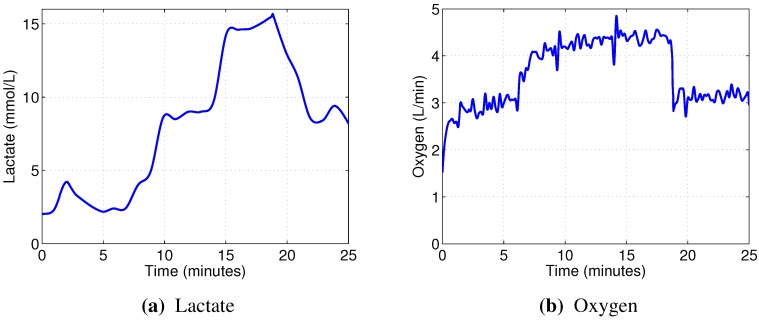
Physiological parameters over time for Subject 2. The graphs are interpolated; blood lactate concentration was measured every minute, and oxygen uptake was measured every 10 s.

Figure 3 presents the time courses of recorded physiological indicators for Subject 2, which are typical for our experiment. Lactate remains relatively low during Phase 1, then starts increasing rapidly throughout Phase 2, until the point where the test subject can no longer maintain the cadence, and then drops off again during Phase 3. In the meantime, oxygen uptake generally follows the load with some delay.

**Figure 4:**
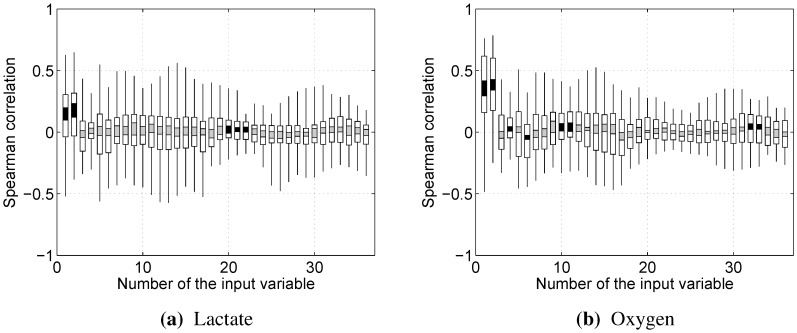
Spearman correlations between the input variables and physiological indicators. Black bars indicate average correlation significantly different from zero (Student’s *t*-test, p<0.05). Every segment corresponds to 0.2 quantiles. Variables and their numbers were defined in Section 2.5.

Figure 4 presents the Spearman correlations between the 36 input variables and the physiological indicators across all test subjects and muscles. It is notable that RMS (1) and dRMS (2) are the only variables that show a clear positive trend in relationship to both blood lactate concentration and oxygen uptake. For the other variables, including the median power frequency (13), there are a few cases with strong positive and negative trends (thin black lines above 0.5 or below −0.5), but they do not apply to most datasets (white and black/gray bars).

**Figure 5:**
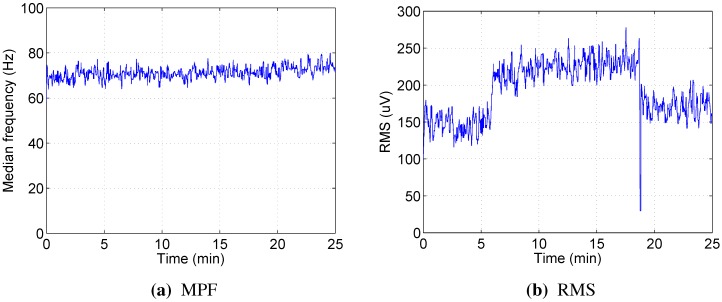
Time courses of the median power frequency and the normalized RMS for right vastus lateralis of Subject 2.

Figure 5 illustrates this by presenting time courses of median EMG power frequency (Figure 5a) and RMS (Figure 5b) of right vastus lateralis muscle for Subject 2, which are, again, typical for our experiments. It can be seen that MPF, usually considered to be indicative of localized muscle fatigue, does not exhibit any downward trend and could be even slightly increasing. Meanwhile, the RMS of EMG signal appears to follow the load closely, while the variation due to fatigue is less obvious, but still evident.

### 3.2. Regression Models to Predict Oxygen Uptake and Blood Lactate Concentration

#### 3.2.1. Linear Models

**Figure 6:**
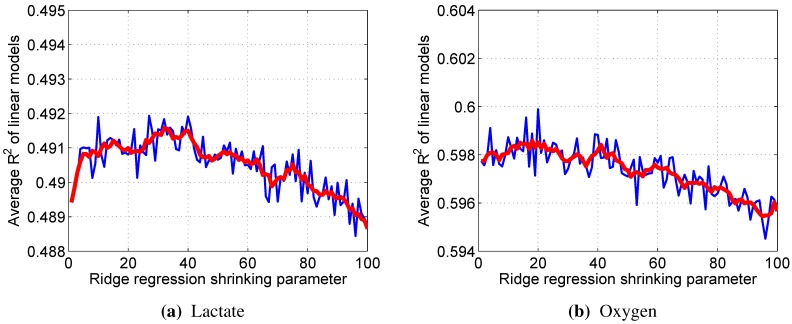
Raw (blue) and smoothed (red) average $R^{2}$ values, obtained with different shrinkage parameter λ.

Linear models with Tikhonov’s (ridge) regularization require determining shrinkage parameter λ beforehand. For this purpose, every integer value from 1–100 was tested with each available dataset, noting the resultant $R^{2}$ values. These values were then averaged across the datasets and smoothed to obtain the graphs in Figure 6. The λ with which the best average $R^{2}$ was obtained (32 for lactate, 18 for oxygen) was then used in further calculations.

Linear models to predict blood lactate concentration and oxygen uptake were built for every muscle of each subject. Additionally, the same calculations were performed with data from all subjects combined into a single set. The results, which are averages obtained by performing 10-fold cross-validation, can be seen in Tables Table 1 and Table 2.

**Table 1:** The coefficient of determination $R^{2}$ for subject-specific, as well as general linear regression models for predicting blood lactate concentration.

Subject	*Rectus Femoris*		*Vastus Lateralis*		*Semitendinosus*		*Biceps Femoris*	
	Left	Right	Left	Right	Left	Right	Left	Right
1	0.577	0.379	0.847	0.494	0.454	0.546	0.671	0.679
2	0.466	0.364	0.355	0.576	0.442	0.300	0.236	0.360
3	0.512	0.216	0.480	0.265	0.257	0.183	0.293	0.347
4	0.469	0.565	0.381	0.324	0.412	0.367	0.451	0.461
5	0.589	0.515	0.726	0.558	0.492	0.495	0.614	0.411
6	0.552	0.537	0.517	0.463	0.304	0.265	0.364	0.605
7	0.501	0.567	0.731	0.316	0.654	0.833	0.670	0.659
8	0.631	0.591	0.890	0.714	0.368	0.518	0.730	0.710
9	0.486	0.535	0.508	0.407	0.396	0.381	0.402	0.392
All	0.284	0.218	0.195	0.098	0.163	0.244	0.074	0.134

**Table 2:** The coefficient of determination $R^{2}$ for subject-specific, as well as general linear regression models for predicting oxygen uptake.

Subject	*Rectus Femoris*		*Vastus Lateralis*		*Semitendinosus*		*Biceps Femoris*	
	Left	Right	Left	Right	Left	Right	Left	Right
2	0.825	0.760	0.703	0.786	0.618	0.562	0.513	0.550
4	0.541	0.706	0.475	0.654	0.742	0.707	0.460	0.756
5	0.659	0.596	0.703	0.632	0.569	0.374	0.503	0.364
6	0.673	0.618	0.556	0.523	0.450	0.308	0.443	0.597
7	0.643	0.654	0.410	0.532	0.526	0.577	0.500	0.620
8	0.602	0.552	0.810	0.731	0.464	0.594	0.809	0.745
All	0.603	0.518	0.426	0.581	0.528	0.612	0.373	0.504

Generally, subject-specific models for oxygen uptake had somewhat higher $R^{2}$ than the ones predicting blood lactate concentration (0.598 average $R^{2}$ *vs.* 0.491), although the performance of linear models was quite variable in both cases. Also notable is significant degradation in blood lactate concentration prediction accuracy when the datasets from different subjects were combined, while the accuracy of oxygen uptake prediction remained more or less the same.

**Figure 7:**
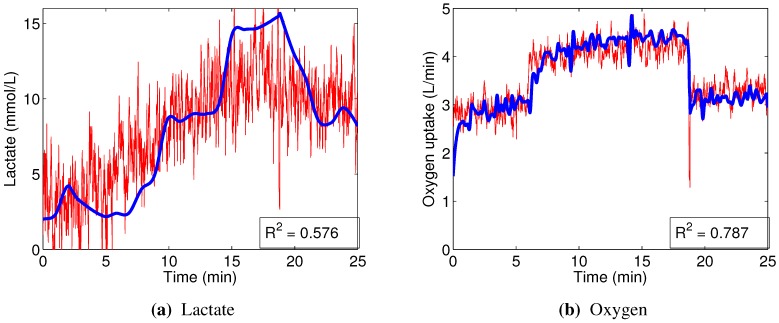
An example of predictions (red), compared to the interpolated ground truth (blue), made by the linear regression models, built using data from right vastus lateralis of Subject 2.

A graphical example of the performance of typical subject-specific linear models can be seen in Figure 7. While the predictions are quite noisy, in the case of oxygen uptake, they follow the general trends in the data quite well. The success of predicting blood lactate concentration, however, is limited: while the prediction graph reflects an increase in the concentration, the structure of this increase is not closely followed.

#### 3.2.2. Regression Random Forests

Regression models to predict $VO_{2}$ and blood lactate concentration were also built using random forests. For these models, the coefficient of determination $R^{2}$ was estimated using the out-of-bag data.

Table 3 presents the coefficient of determination $R^{2}$ of random forests built from subject-specific and combined datasets to predict blood lactate concentration. Similarly, Table 4 presents the results for models predicting oxygen uptake.

**Table 3:** The coefficient of determination $R^{2}$ for subject-specific, as well as general random forest regression models for predicting blood lactate concentration.

Subject	*Rectus Femoris*		*Vastus Lateralis*		*Semitendinosus*		*Biceps Femoris*	
	Left	Right	Left	Right	Left	Right	Left	Right
1	0.929	0.956	0.893	0.909	0.944	0.888	0.895	0.958
2	0.884	0.802	0.831	0.789	0.838	0.886	0.795	0.831
3	0.902	0.865	0.868	0.882	0.836	0.844	0.851	0.843
4	0.834	0.833	0.904	0.839	0.880	0.834	0.850	0.907
5	0.899	0.942	0.864	0.890	0.872	0.882	0.873	0.843
6	0.902	0.869	0.795	0.836	0.885	0.873	0.777	0.881
7	0.893	0.928	0.915	0.918	0.904	0.795	0.958	0.906
8	0.876	0.979	0.813	0.934	0.880	0.924	0.872	0.901
9	0.853	0.863	0.815	0.804	0.832	0.870	0.809	0.818
All	0.798	0.824	0.788	0.824	0.774	0.836	0.853	0.805

**Table 4:** The coefficient of determination $R^{2}$ for some subject-specific, as well as general random forest regression models for predicting oxygen uptake.

Subject	*Rectus Femoris*		*Vastus Lateralis*		*Semitendinosus*		*Biceps Femoris*	
	Left	Right	Left	Right	Left	Right	Left	Right
2	0.974	0.925	0.876	0.859	0.960	0.968	0.876	0.855
4	0.860	0.868	0.934	0.831	0.926	0.896	0.931	0.941
5	0.907	0.914	0.899	0.878	0.886	0.884	0.817	0.824
6	0.934	0.891	0.848	0.868	0.921	0.917	0.814	0.884
7	0.918	0.832	0.873	0.875	0.910	0.864	0.902	0.903
8	0.879	0.960	0.860	0.967	0.882	0.944	0.900	0.905
All	0.907	0.921	0.932	0.886	0.888	0.903	0.897	0.867

The random forest models showed very good predictive power ($R^{2}$ mostly above 0.8 for both $VO_{2}$ and blood lactate concentration). On average, random forest models performed significantly better than their linear counterparts (twin-tailed *t*-test, $p<10^{-33}$ for lactate, $p<10^{-21}$ for oxygen, for subject-specific datasets). The performance of the models did not show large deterioration when using the combined datasets for neither blood lactate nor oxygen uptake. However, just as with the linear models, the models predicting oxygen uptake performed slightly better (0.895 *vs*. 0.871 average $R^{2}$) than the ones predicting blood lactate concentration.

**Figure 8:**
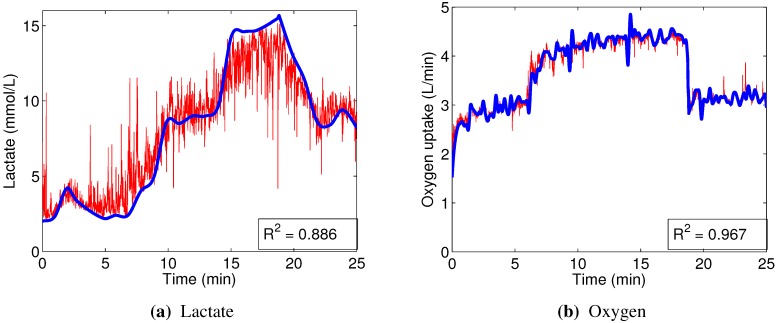
An example of out-of-bag predictions (red), compared to the interpolated ground truth (blue), made by the random forest regression models, built using data from right vastus lateralis of Subject 2.

An example of the performance of random forest models can be seen in Figure 8. Similarly to Figure 7, these sample models were built using data from the right vastus lateralis of Subject 2. The predictor was capable of following the $VO_{2}$ ground truth curve very closely, even in what could be regarded as small details, with only occasional minute deviations (Figure 8). The models to predict blood lactate concentration were less accurate, but they showed a clear improvement over their linear counterparts, correctly representing all significant changes in the trend.

### 3.3. Evaluation of the Significance of Parameters

While the accuracy of the models is important, it is equally important to build an understanding of which of the input variables contribute the most to the predictions that the models make. One very basic measure of that is correlation between input and output variables, which was already investigated in Section 3.1. There, we saw that, apart from RMS and dRMS measures, no other input variables produced particularly strong correlations with either lactate concentration or oxygen uptake.

**Figure 9:**
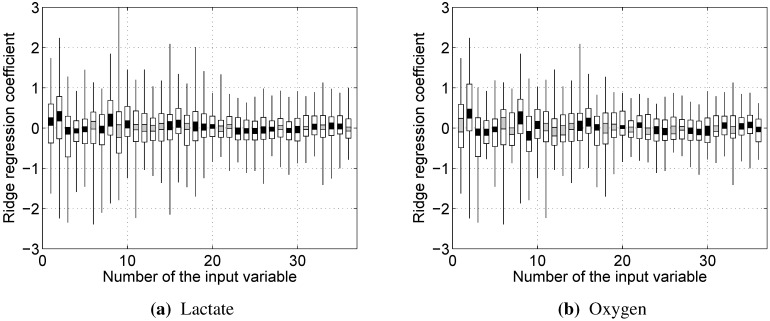
The distribution of input variable weights in linear regression models over all subject-specific datasets. Each segment of a bar represents an interval of 0.2 quantiles. Black bars represent input variables, for which the mean weight is significantly (p<0.05) different from zero. Variables and their numbers were defined in Section 2.5.

Since z-scores were used when building linear models, the importance of a particular variable can be assessed by its weight in the model. The distribution of these weights is provided in Figure 9. It can be seen that the weights are in almost all cases spread around zero, with the notable exceptions of RMS (1, lactate only), dRMS (2), kurtosis (8) and 10th percentile (9, oxygen only). A few other variables have mean weights significantly (Student’s *t*-test, p<0.05) different from zero (shown by black bars in Figure 9); however, their weights are not particularly large.

Most implementations of random forests provide built-in methods to assess the importance of input variables for the final model. Variable importance is estimated by measuring the degradation of prediction for out-of-bag data when the variable gets randomly scrambled. If the deterioration is large, it can be assumed that the variable is important; similarly, small deterioration means variable importance is low.

**Figure 10:**
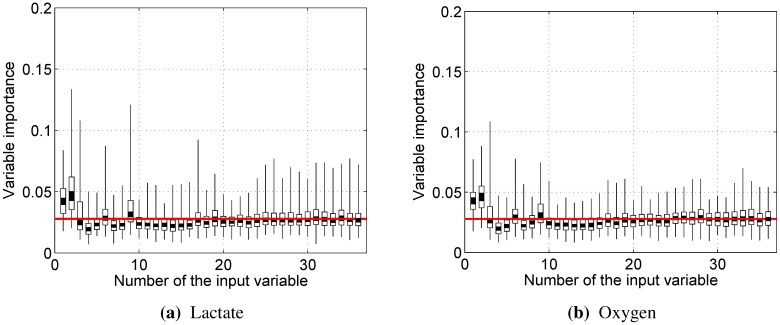
The distribution of the input variable importance for random forest models. Each segment of a bar represents a step of 0.2 quantiles. The red line represents the expectation if all variables were equally important. Variables and their numbers were defined in Section 2.5.

Differently from correlations, this measure is not limited to linear relationships; therefore, it is capable of reflecting more complex interactions. A graph of the distribution of variable importance over all random forest models is presented in Figure 10. The red line signifies the expectation if all variables were equally important.

Only RMS (1), dRMS (2) and 10th percentile (9) show importance that is higher than expected. The last result might be seen as slightly surprising, but it should be noted that 90th percentile (17) is also valued more than other percentile data (10–16), suggesting that the behavior of the tails of the EMG power distribution is the important factor. However, it must be noted that the success of random forest models relies on the whole set of input variables, even if some of them are only moderately important on their own.

It is also worthwhile to point out that instantaneous frequency (3), mean frequency (5) and median frequency (13), commonly used as fatigue indicators, were not particularly important for predicting neither blood lactate concentration nor oxygen uptake.

## 4. Discussion

The median power frequency, which is regarded as one of the best indicators for muscle fatigue in high static load experiments, was not informative in dynamic load exercises using our protocol. Moreover, different patterns of MPF change were observed for different subjects participating in the experiment. The decrease over time was observed only for a few muscles in a few participants, and in most cases, MPF remained stable or even increased slightly as the test progressed. This agrees with studies from Knaflitz [17], Jansen [18] and Tenan [14] and supports the belief that the limiting factor for this type of exercise is the inability of cardiovascular and respiratory systems to supply enough oxygen to the muscles, not local muscle fatigue.

The linear models designed to predict oxygen uptake were rather successful in their capacity to track the general change in dynamics. Predicting blood lactate concentration, on the other hand, was a more difficult task. In particular, the general linear models, built from the combined data, barely outperformed the naive predictor (mean of the training set), as evidenced by the coefficients of determination $R^{2}$ ranging between 0.05 and 0.3. This, combined with significantly higher accuracy of nonlinear models in general (as shown in Section 3.2.2), strongly suggests that the relationships between the input variables that we used and blood lactate concentration are non-linear.

In contrast, the non-linear random forest models built to predict blood lactate concentration showed very good performance, evidenced by the high out-of-bag prediction accuracy. Even better results were obtained when predicting oxygen uptake. Those models were able to reflect not only the major changes in oxygen consumption, but also the minor details.

Almost every model built showed rather strong reliance on the RMS and/or dRMS features. This result is not unexpected for $VO_{2}$ prediction, since they are directly linked and closely follow the load a cyclist is experiencing. However, the same being true for prediction of lactate accumulation suggests that changes in RMS and dRMS also reflect the exertion of the cyclist to some extent. More interestingly, our results suggest that, while neither mean nor median power frequency were important in predicting blood lactate concentration or oxygen uptake, the tails of the EMG power distribution carry useful information to predict these two physiological parameters.

Having said that, it must be understood that the main goal of our experiments was not the identification of a simple relationship between blood lactate concentration or oxygen uptake and one or two input variables, characterizing the EMG signal. Indeed, the success of random forest models using a large set of moderately-important variables suggests that this relationship is rather complex and non-linear.

As already discussed in Section 2.5, our results rely on the assumption of cyclic stationarity of the extracted sEMG segments, as outlined in a 2001 article by Bonato *et al*. [9]. While this assumption allows meaningful comparison between these segments, employing alternative, more complicated methods of time-frequency analysis, such as continuous wavelet transform, could yield even better results without assuming any type of stationarity.

Due to the relatively low number of test subjects, it would be quite hard to draw any firm conclusions on within-group differences in the results, e.g., between male and female participants. Having said that, we did not observe any strong gender or age biases in either the performance of the subjects during the tests or the results we obtained during the analysis.

Random forests show a great potential for analyzing EMG data and could be used successfully in this context. In particular, more advanced time-frequency domain techniques could be used when extracting the spectral information from EMG signals, which would likely further increase the prediction accuracy that random forests were able to achieve. In addition, EMG signal analysis needs not be limited to the frequency or time-frequency domain, as useful information could be extracted from multichannel sEMG recordings by analyzing the timing of certain events, e.g., muscle activation and deactivation. This particular approach could also provide some insight into the changes in cycling kinematics as the exercise progresses and fatigue increases.

## 5. Conclusions

1. Neither mean nor median frequency provide more information than other selected variables for predicting oxygen uptake and blood lactate concentration. Variable importance values computed for both linear and non-linear models substantiate this conclusion.
2. The statistically-significant difference in performance observed between the linear and nonlinear models and the relatively high prediction accuracy obtained from the nonlinear models indicate the existence of nonlinear relations between the set of independent variables and dependent variables in the studied datasets. Therefore, non-linear models should be used to predict oxygen uptake and blood lactate concentration from the EMG power spectrum characteristics.
3. The non-linear models provided high prediction power with both subject-specific and general datasets. This was not true for the linear models. These results confirm the higher ability of the non-linear models, not only to adapt to, but also to generalize over subject-specific variations manifesting themselves in values of the EMG power spectrum characteristics.
4. Very similar results obtained from the models built using data from different subjects, in spite of variations in the testing protocol, confirm the consistency of the parameter set used to build the models.
